# Sandwich-Structured, Hydrophobic, Nanocellulose-Reinforced Polyvinyl Alcohol as an Alternative Straw Material

**DOI:** 10.3390/polym13244447

**Published:** 2021-12-18

**Authors:** Chun-Tu Chou, Shih-Chen Shi, Chih-Kuang Chen

**Affiliations:** 1 Department of Mechanical Engineering, National Cheng Kung University (NCKU), Tainan 70101, Taiwan; elephant412@gmail.com; 2 Department of Materials and Optoelectronic Science, National Sun Yat-sen University, Kaohsiung 80424, Taiwan; chihkuan@mail.nsysu.edu.tw

**Keywords:** sandwich structure, hydrophobic, polyvinyl alcohol, cellulose, straw

## Abstract

An environmentally friendly, hydrophobic polyvinyl alcohol (PVA) film was developed as an alternative to commercial straws for mitigating the issue of plastic waste. Nontoxic and biodegradable cellulose nanocrystals (CNCs) and nanofibers (CNFs) were used to prepare PVA nanocomposite films by blade coating and solution casting. Double-sided solution casting of polyethylene-glycol–poly(lactic acid) (PEG–PLA) + neat PLA hydrophobic films was performed, which was followed by heat treatment at different temperatures and durations to hydrophobize the PVA composite films. The hydrophobic characteristics of the prepared composite films and a commercial straw were compared. The PVA nanocomposite films exhibited enhanced water vapor barrier and thermal properties owing to the hydrogen bonds and van der Waals forces between the substrate and the fillers. In the sandwich-structured PVA-based hydrophobic composite films, the crystallinity of PLA was increased by adjusting the temperature and duration of heat treatment, which significantly improved their contact angle and water vapor barrier. Finally, the initial contact angle and contact duration (at the contact angle of 20°) increased by 35% and 40%, respectively, which was a significant increase in the service life of the biodegradable material-based straw.

## 1. Introduction

Approximately 8 million tons of plastic waste has been estimated to be discarded into oceans annually; plastic packaging, which constitutes half of this global aggregate, is the primary source [1]. Plastic straws, which account for approximately 1% of this share, are typically prepared using polypropylene (PP) and low-density polyethylene (LDPE). While PP offers adequate resistance to acidic/alkaline foods and heat (melting temperature, T_m_ ≈ 165 °C) [2], LDPE straws exhibit poor heat resistance (T_m_ ≈ 101 °C) [3] and can consequently be used only for beverages under 65 °C. PP and LDPE are extensively used because of their various advantages such as low manufacturing costs, low weight, waterproofness, and ease of transport. However, these nondegradable plastics are known to pollute ecological environments, as evidenced by concerning discoveries such as the occupation of bird habitats by marine debris [4]. More than 5 trillion plastic waste items, weighing more than 250,000 tons, are currently floating in oceans [5], indicating the detrimental effects of plastic waste on ecological environments. Various efforts are being made to reduce the amount of nondegradable plastic waste.

Polyvinyl alcohol (PVA) is a water-soluble, biodegradable, transparent, chemically resistant, and biocompatible polymer that is present in everyday products such as glues. PVA can be produced by alkaline hydrolysis of polyvinyl acetate [6,7]. PVA films are currently manufactured primarily by solution casting [6]. Processes such as melt processing and blown film extrusion have also been recently developed. However, the predominant issue in the blown film extrusion of PVA involves its comparable melting point (T_m_ ≈ 228 °C) [8] and degradation temperature (≈225 °C) [6]. Several attempts have been made to manufacture PVA by solution casting and by adding fillers such as chitosan [9], graphene [10], silver nanoparticles [11], and date palm leaf fibers [12] to yield materials with significantly enhanced mechanical and thermal properties. The high polarity of PVA and its ability to be processed in aqueous solutions make it a decent candidate for mixing with natural polymers to create biodegradable composite materials.

Nanocellulose, a novel biodegradable material, can be categorized into cellulose nanocrystals (CNCs) and cellulose nanofibers (CNFs). In both cases, lignin and hemicellulose are removed from lignocellulose, which is followed by different preparation processes. In addition to being used as packaging materials for food [13], CNCs and CNFs exhibit excellent properties such as abundant intramolecular and intermolecular hydrogen bonds. Therefore, CNCs and CNFs with Young’s moduli of 110–200 GPa [14,15] and 130–150 GPa [16,17], respectively, have been realized. With respect to the penetration of water molecules, the compact structures of CNCs and CNFs can increase the path length as well as the difficulty of penetration [18]. In addition, CNCs and CNFs offer additional advantages such as high aspect ratios, straightforward surface modification, and nontoxicity [19,20,21,22]. They are safer and less risky to the environment compared with other nanoparticles [23].

The addition of CNCs or CNFs to PVA can lead to stronger H-bonding because of the presence of numerous hydroxyl groups (–OH) in PVA, CNCs, and CNFs. This addition is known to significantly improve the mechanical properties [21,22,24,25,26] and thermal stabilities [25,26,27,28] of the resulting composites and reduce their water vapor permeability (WVP) and water uptake [21,24], so PVA can be more widely used in product applications. Owing to the water-soluble nature of PVA and its excellent film-forming properties in aqueous solvents, PVA films can be readily manufactured under mild conditions. However, PVA exhibits poor resistance to humid environments owing to its hydrophilicity and the presence of numerous hydroxyl groups. Water molecules can readily penetrate PVA films under humid environments and cause swelling, which deteriorates its physical and mechanical properties. Several approaches have been adopted to overcome this limitation, such as the addition of a certain amount of graphene oxide (GO), which can slightly reduce the swelling effect. However, the presence of –COOH and –OH groups on the GO surface, which can form hydrogen bonds with water, prevents significant enhancement of the hydrophobicity of PVA [29]. Crosslinking PVA with aldehydes is another popular strategy. For example, glyoxal [30,31] and glutaraldehyde [32] can crosslink PVA to form a 3D structure and increase its water resistance [33]. However, the brittleness of the crosslinked PVA and the presence of unreacted crosslinking agents prevent the widespread use of PVA as a green material [33].

Polyethylene-glycol–poly(lactic acid) (PEG–PLA) is a block copolymer that is commonly used as a drug carrier [34,35]. PLA is a biodegradable, biocompatible, nontoxic polymer with excellent mechanical properties [34]; therefore, the U.S. Food and Drug Administration has approved its use in drug carriers, medical materials, and tissue engineering applications. However, the hydrophobicity of PLA and its long degradation time restrict its application [36]. PEG, on the other hand, exhibits hydrophilicity, flexibility, biocompatibility, and antiphagocytosis against macrophages [34]. The polymerization of PEG and PLA can extend the residence time of drugs in human bodies, which can prevent its ingestion by macrophages. Additionally, PEG–PLA is hydrophilic on the PEG end and hydrophobic on the PLA end, which permits bonding between the hydrophilic end and the hydrophilic PVA, with the hydrophobic end being connected to the hydrophobic neat PLA.

To reduce the amount of nondegradable plastic waste from straw, the present study was aimed at resolving the hydrophilic characteristics of PVA. The addition of CNCs or CNFs to PVA can reduce its WVP and water uptake. The performance of sandwich-structured PVA-based hydrophobic composite films, which were prepared using hydrophobic and biodegradable PEG–PLA and neat PLA, was compared to that of commercial PP straws. A list of abbreviations used in this study is presented in Table A1 of Appendix A.

## 2. Materials and Methods

### 2.1. Materials

#### 2.1.1. PVA + CNC/CNF Nanocomposite Films

PVA (BF-26; MW, ≈14,400; degree of hydrolysis, 98.5–99.2%) was purchased from Chang Chun Petrochemical (Kaohsiung, Taiwan) for the synthesis. The CNC and CNF fillers for PVA were purchased from CelluForce (CelluForce NCC, Montreal, QC, Canada) and Nanografi (Thuringia, Germany), respectively.

#### 2.1.2. Hydrophobic Materials

Hydrophobic materials including PEG (MW, 1900–2200) and lactide (LA; purity > 98%; MW, ≈144.13) were purchased from Sigma-Aldrich (Saint Louis, MO, USA). PLA (2003D), the other hydrophobic material, was purchased from NatureWorks (Minnetonka, MN, USA).

### 2.2. Methods

#### 2.2.1. Preparation of PVA + CNC/CNF Nanocomposite Films

**Figure 1:**
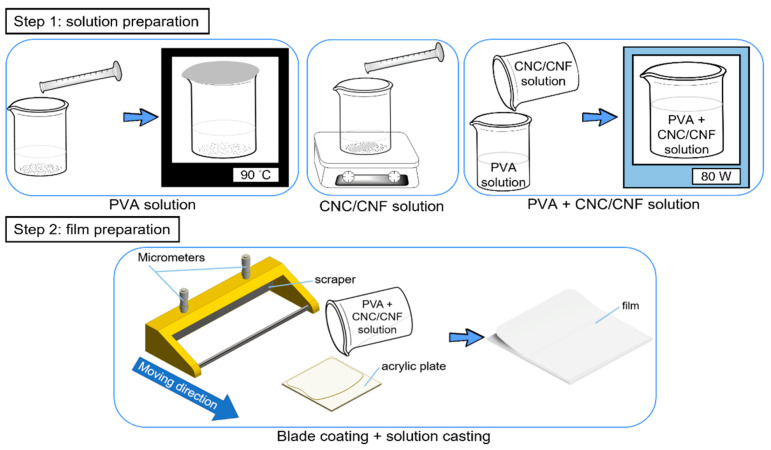
Preparation of PVA + CNC/CNF nanocomposite films.

PVA + CNC/CNF nanocomposite films were prepared by blade coating (1806/300, ±2 μm, BEVS, Guangzhou, China) and solution casting. The details of this process are illustrated in Figure 1. The protocol can be divided into two steps.

The first step involved the preparation of PVA + CNC/CNF solutions. To prepare the PVA solution, an appropriate amount of PVA particles (10 wt%) was added to a beaker containing 50 mL of deionized water, and it was subsequently sealed using aluminum foil. The beaker was placed in an oven (DH400, ±1 °C, DENGYNG, New Taipei, Taiwan) at 90 °C for 1 h and subsequently removed and allowed to cool at a temperature of 23 ± 2 °C and humidity (RH) of 45 ± 5% for 1 h. Next, to prepare the CNC/CNF solution, CNCs/CNFs with different weight percentages were added to 20 mL of deionized water and stirred using an electromagnetic stirrer (PC-420D, ±5% rpm, CORNING, New York, USA) of 300 rpm at 23 ± 2 °C. Finally, the CNC/CNF solutions were poured into the PVA solution, mixed, and subsequently agitated using an ultrasonic cleaning machine (DC80H, DELTA ULTRASONIC, New Taipei, Taiwan) for 10–20 min. The resulting solutions were stored at 23 ± 2 °C for 3 h.

The second step involved the preparation of PVA + CNC/CNF nanocomposite films. The corresponding prepared solutions were poured onto 2 mm thick acrylic plates. Micrometers were adjusted to a height of 3.5 mm to drive a scraper to strip off excess solution. Films were removed from the acrylic plates after 24 h of evaporation of the solutions.

#### 2.2.2. Preparation of Hydrophobic Materials

**Figure 2:**
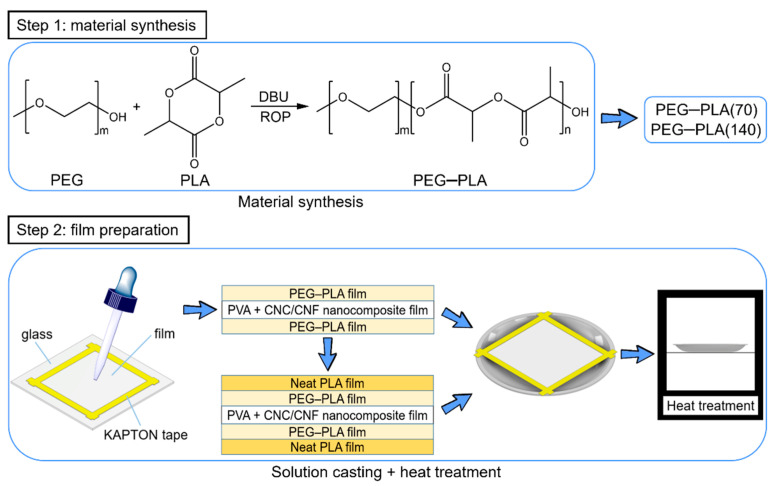
Preparation of hydrophobic materials and sandwich-structured PVA-based hydrophobic composite films.

The hydrophobic materials were prepared by polymerization and subsequently subjected to solution casting and heat treatment for producing the hydrophobic films. A detailed experimental flowchart, which consists of two steps, is illustrated in Figure 2.

The first step involved the polymerization of PEG–PLA. PEG and DBU (1,8-diazabicyclo [5.4.0] undec-7-ene; MW, 152.24; Sigma-Aldrich) were used as an initiator and catalyst, respectively, for the ring-opening polymerization (ROP) of LA. This was achieved by controlling the PEG:DBU and LA:DBU ratios. PEG–PLA(70) and PEG–PLA(140) with different LA chain lengths were polymerized in this study; the experimental parameters are listed in Table 1.

PEG, LA, and various solvents were purified to remove water prior to the polymerization of PEG–PLA. Solvent distillation was employed to heat dichloromethane (DCM, HPLC grade; CAS 75-09-2; DUKSAN, Gyeonggi, Korea) and ethyl acetate (EA; CAS 141786, Macron, NJ, USA) to 60 °C and 90 °C, respectively. To purify PEG, 200 mg of PEG was added to a volumetric flask, dissolved in 1 mL of DCM, and drained for 15 min; the resulting solution was dissolved in 1 mL of toluene (CAS 108-88-3; DUKSAN, Gyeonggi, Korea) and drained for 15 min. These aforementioned steps were repeated three times. Finally, 1 mL of DCM was used to dissolve PEG, and the resulting solution was drained for 15 min; this step was repeated twice. For purifying LA, 5 g of LA was added to 5 mL of EA and dissolved for 20–30 min in an oil bath at 65–70 °C under N_2_. After the LA was completely dissolved, the solution was placed in an ice bath for 20–30 min, and excess EA was removed with a syringe. Finally, a water pump (SHB-III, Zhengzhou Great Wall Scientific Industrial and Trade, Zhengzhou, China) was used to remove EA for 20–30 min. It is worth noting that EA was used twice (4 mL and 3 mL, respectively) to dissolve LA until the pump removed the excess EA.

For polymerization, PEG was dissolved in 1 mL of DCM. Various amounts of LA were subsequently added to this mixture. The amounts of PEG, LA, and DBU used in this study are listed in Table 1. After the complete dissolution of LA, DBU-containing DCM was added to this mixture and stirred at 30 °C for 2 h. In this step, 2 mL and 2.5 mL of DCM were required for PEG–PLA(70) and PEG–PLA(140), respectively. The polymer viscosity increased during polymerization because of an increase in the molecular weight. Furthermore, a water pump was used to remove the solvent containing PEG, LA, and DBU for 5 min. During this process, the stir bar was rotated to prevent the polymer from being removed. The polymer was subsequently vacuumed for 30 min. Moreover, DCM was added to completely dissolve PEG–PLA by stirring for 30 min; essentially, 3 mL and 5 mL of DCM were required for PEG–PLA(70) and PEG–PLA(140), respectively. Diethyl ether (CAS 60-29-7; DUKSAN, Gyeonggi, Korea) was added to a 250 mL Erlenmeyer flask, with 30 mL and 50 mL being required for PEG–PLA(70) and PEG–PLA(140), respectively. The completely dissolved PEG–PLA solution was poured into the Erlenmeyer flask and the glass lid was covered; a parafilm was subsequently adhered and the flask was placed in a refrigerator for 12 h. When the eluent was poured out, a white crystalline precipitate was produced, which was placed on a rotary evaporator (3–5 times) to remove residual solvents.

The second step involved the preparation of hydrophobic films. Chloroform was used to separately dissolve PEG–PLA(1 wt%) and neat PLA (1 wt%). KAPTON tape was used to adhere the investigated films onto a glass layer. Air bubbles were sufficiently squeezed out prior to adhering this tape. Sandwich-structured PVA-based PEG–PLA and neat PLA films were prepared by solution casting using the aforementioned chloroform solutions. Subsequently, a dropper was used to place these solutions onto the test films, and the solvent was evaporated for 50 min. The KAPTON tape was subsequently removed. Upon the completion of solution casting on one of its sides, the film was overturned (180°) for solution casting on its other side.

Two types of sandwich-structured PVA-based hydrophobic composite films were prepared in this study. The first was prepared via double-sided solution casting of PEG–PLA with a PVA composite film as the substrate; the other was prepared by double-sided solution casting of neat PLA on this aforementioned PVA composite film containing PEG–PLA.

Prior to the heat treatment, KAPTON tape was used to adhere the test film onto a stainless steel plate, which was placed in an oven at different temperatures and durations. The temperatures, durations, and notations pertaining to the heat treatment are listed in Table 2.

### 2.3. Characterization

#### 2.3.1. X-ray Diffraction

X-ray diffraction (XRD) analysis was performed using an X-ray diffractometer (Ultima IV, Rigaku, Tokyo, Japan) to assess possible damage to the materials by the employed processes. The variations in the crystallization peaks and grain sizes of the PVA + CNC/CNF nanocomposite films were investigated. The grain size can be obtained using the Scherrer formula [37,38]. Additionally, the possible presence of PEG and PLA crystallization peaks in the heat-treated hydrophobic materials was examined. An angular scanning range of 5–40° was employed with a step of 0.01°. Two samples were prepared for the XRD analysis.

The crystal sizes were estimated using the Scherrer equation as shown in Equation (1):

D = Kλ/βcos θ(1)

where D (nm) is the crystal dimension perpendicular to the diffracting (hkl) planes, K (0.89) is the Scherrer constant, λ (0.154056 nm) is the wavelength of X-ray radiation, β (rad) is the full width at half maximum of the diffraction peak, and θ (°) is the diffraction angle.

#### 2.3.2. Contact Angle Analysis

Contact angles were determined in accordance with the ASTM D7334 protocol using a contact angle goniometer (100SB, ±0.1°, Sindatek, New Taipei, Taiwan). The pendant drop method was employed, and built-in software was used to calculate the contact angles. Five samples were prepared for the contact angle analysis. Contact angles less than or greater than 90° indicate hydrophilicity and hydrophobicity, respectively. These measurements were conducted to examine the variations in the contact angles of the sandwich-structured PVA-based hydrophobic composite films over time and to immediately measure the changes in the contact angles of the PVA + CNC/CNF nanocomposite films. Approximately 3.9–4.1 μL of deionized water was added to the surface of the prepared films. The specimens were fixed to a thin plate with double-sided tape prior to the measurements to ensure that the films were horizontal.

#### 2.3.3. Water Vapor Permeability

**Figure 3:**
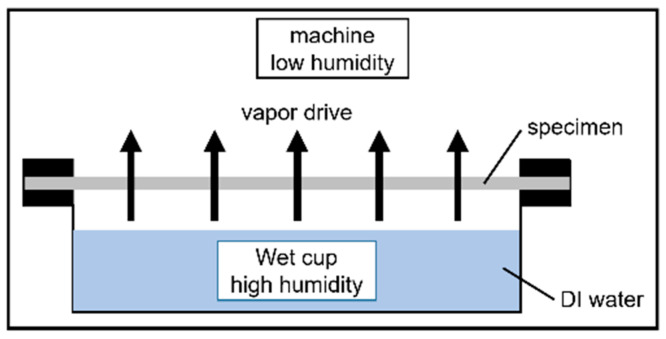
ASTM E96/E96M-16 upright cup and the wet cup method.

Water vapor permeability was determined in accordance with the ASTM E96/E96M-16 protocol using a constant temperature and humidity machine (DE80, ±0.5 °C, ±3% RH, DENGYNG, New Taipei, Taiwan). Using the upright cup and the wet cup method, water vapor was transmitted from the wet cup (high humidity) to the atmosphere inside the machine (low humidity), as shown in Figure 3. The humidity and temperature of this apparatus were estimated to be 50 ± 2% and 23 ± 1 °C, respectively. The WVP was monitored to determine the changes in in the WVP of the PVA + CNC/CNF nanocomposite films and to determine the possible effects of heat treatment of the sandwich-structured PVA-based hydrophobic composite films PEG and PLA crystals on the WVP.

The equation of WVP is shown in Equation (2):

WVP = WVTR·y/ΔP(2)

where WVTR is the slope of the change in weight over time, y (mm) is the thickness of the specimen, and ΔP is the pressure difference across the interior and exterior surfaces of the specimen (1.40 kPa).

WVTR in Equation (2) is shown in Equation (3):

WVTR = G/tA(3)

where G (g) is the change in the weight of the WVP cup containing the specimen, t (h) is the test duration, and A (m^2^) is the area of the specimen.

#### 2.3.4. Differential Scanning Calorimetry (DSC)

Thermal property analysis was performed using a simultaneous thermal analyzer (STA 449 F3 Jupiter, <0.5 µW, NETZSCH, Bavaria, Germany). Two samples were prepared for the DSC. This test was primarily performed to analyze the glass transition temperatures (T_g_) and crystallinities (X_c_) of the PVA + CNC/CNF nanocomposite films. This enabled the determination of possible interactions between the CNC/CNF and the PVA. Additionally, the cold crystallization temperatures (T_cc_), melting temperatures (T_m_), and X_c_ values of the hydrophobic materials were analyzed, with and without heat treatment for 1 h and 2 h. Samples weighing between 9.5 and 10.5 mg were used. The PVA composite films were heated from 30 to 250 °C, and the hydrophobic materials were heated from 30 to 200 °C; a heating rate of 10 °C/min and a nitrogen atmosphere were used in both scenarios.

The degree of crystallinity of PVA was calculated using Equation (4):

(4) $$X_{c}(\mathrm{PVA})=\Delta H_{m}/w{\Delta H}_{m}^{0}$$

where w is the weight fraction of the PVA matrix in the PVA composites, ΔH_m_ (J/g) is the heat of fusion, and ${\Delta H}_{m}^{0}$ is the heat of fusion of 100% crystalline PVA (161 J/g) [39,40].

The degree of crystallinity of PLA was calculated using Equation (5):

(5) $$X_{c}(\mathrm{PLA})=(\Delta H_{m}-\Delta H_{\mathrm{cc}})/{\Delta H}_{m}^{0}$$

where ΔH_m_ (J/g) is the heat of fusion, ΔH_cc_ (J/g) is the heat of crystallization, and ${\Delta H}_{m}^{0}$ is the heat of fusion of 100% crystalline PLA (93.1 J/g) [41].

#### 2.3.5. Thermogravimetric Analysis (TGA)

Mass spectrometric analysis was performed using a simultaneous thermal analyzer (STA 449 F3 Jupiter, 0.1 µg, NETZSCH, Bavaria, Germany). This analysis can be used to evaluate the dehydration and thermal degradation of polymers by TGA and DTG (derivative thermogravimetry; first derivative of the TGA curve). In addition, the onset (T_onset_), offset (T_offset_), and maximum thermal degradation temperatures (T_max_) can be estimated. TGA was conducted to analyze the variations in the thermal degradation of the PVA + CNC/CNF nanocomposite films to determine the influence of the CNC/CNF and the PVA, and to assess the possible thermal degradation of the hydrophobic materials by the heat treatment. Samples weighing between 9.5 and 10.5 mg were employed. The PVA + CNC/CNF nanocomposite films were heated from 60 to 600 °C, and the hydrophobic materials were heated from 30 to 600 °C; a heating rate of 10 °C/min and a nitrogen atmosphere were employed in both scenarios.

#### 2.3.6. Water Uptake

Water absorption was determined using the aforementioned constant temperature and humidity machine. Water absorption can be used to observe weight changes over time due to certain factors such as swelling. The specimens were first dried at 50 °C for 1 day and subsequently immersed in 300 mL of distilled water at 23 °C. Water absorption was determined by gravimetric analysis every 20 min for 3 h. Sufficient care was taken to ensure that the specimens did not absorb any surface moisture during weighing. The specimens were weighed immediately after being removed from the experimental environment.

The water absorption was calculated using Equation (6):

W = ((W_t_ − W_o_)/W_o_) × 100% (6)

where W_o_ (mg) is the dry weight of the specimen and W_t_ (mg) is the weight at a specific time.

#### 2.3.7. X-ray Photoelectron Spectroscopy (XPS)

Elemental analysis was performed by XPS (PHI 5000 VersaProbe; ULVAC, Kanagawa, Japan). XPS is a surface-analysis technique that enables the deduction of the chemical composition of material surfaces via calculation of their peak areas. XPS was employed to probe the possible presence of chlorine residue on the surface of the heat-treated sandwich-structured PVA-based hydrophobic composite films. Although a chlorine residue specification does not exist in Taiwanese food packaging standards, this test was performed to emphasize the safety of the fabricated films.

## 3. Results and Discussion

### 3.1. PVA + CNC/CNF Nanocomposite Films

#### 3.1.1. X-ray Diffraction

**Figure 4:**
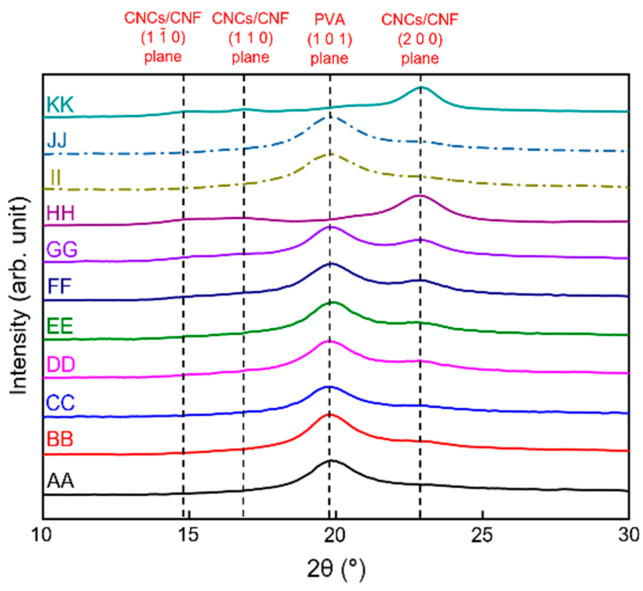
XRD patterns of the PVA + CNC/CNF nanocomposite films. The AA–KK notations are described henceforth. AA: Neat PVA, BB: PVA + 0.5 wt% CNCs, CC: PVA + 1.0 wt% CNCs, DD: PVA + 1.5 wt% CNCs, EE: PVA + 2.0 wt% CNCs, FF: PVA + 2.5 wt% CNCs, GG: PVA + 3.0 wt% CNCs, HH: Neat CNCs, II: PVA + 0.5 wt% CNFs, JJ: PVA + 1.0 wt% CNFs, and KK: Neat CNFs.

XRD was employed to determine the possible damage to the investigated materials by the film preparation processes. The XRD patterns of neat PVA, PVA + CNC/CNF nanocomposite films, neat CNCs, and neat CNFs are shown in Figure 4. A clear crystallization peak was observed for neat PVA at 19.8°, which corresponds to the (1 0 1) plane [39]. The neat CNCs and CNFs exhibited significant peaks at 22.9°, which correspond to the I_β_ (2 0 0) plane of nanocellulose [42]. The increasing addition of CNCs or CNFs to the PVA composite film did not alter the signal intensity exhibited by PVA, indicating that the crystal structure of PVA remained unchanged [43]; however, the peak intensities of the CNCs and CNFs gradually increased.

**Figure 5:**
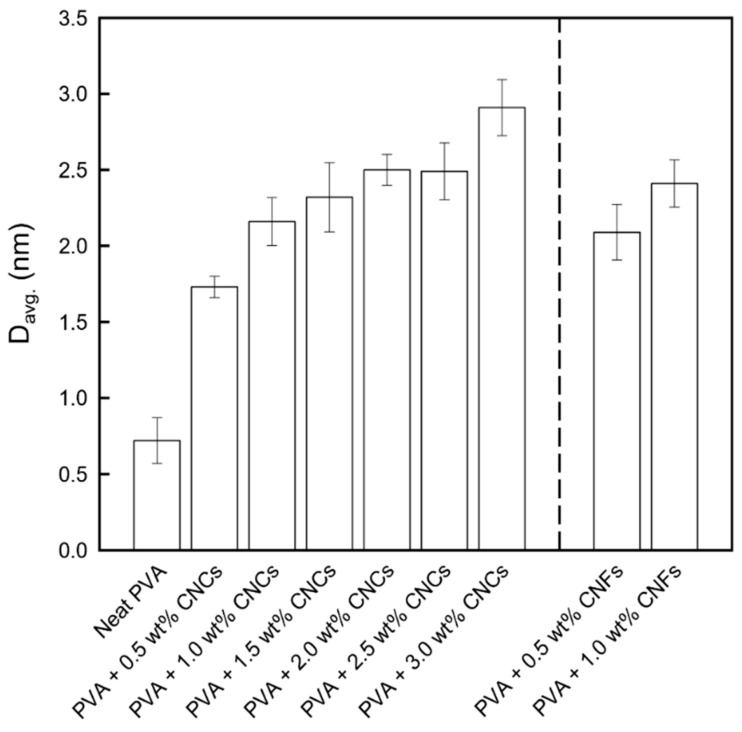
Grain sizes of the PVA + CNC/CNF nanocomposite films.

The crystal grain size can be estimated from the XRD data using the Scherrer formula, as shown in Figure 5. Increasing the weight percentages of the nanocellulose fillers led to an increase in the average crystal grain sizes, which was further verified by DSC analysis.

#### 3.1.2. Contact Angle Analysis

**Figure 6:**
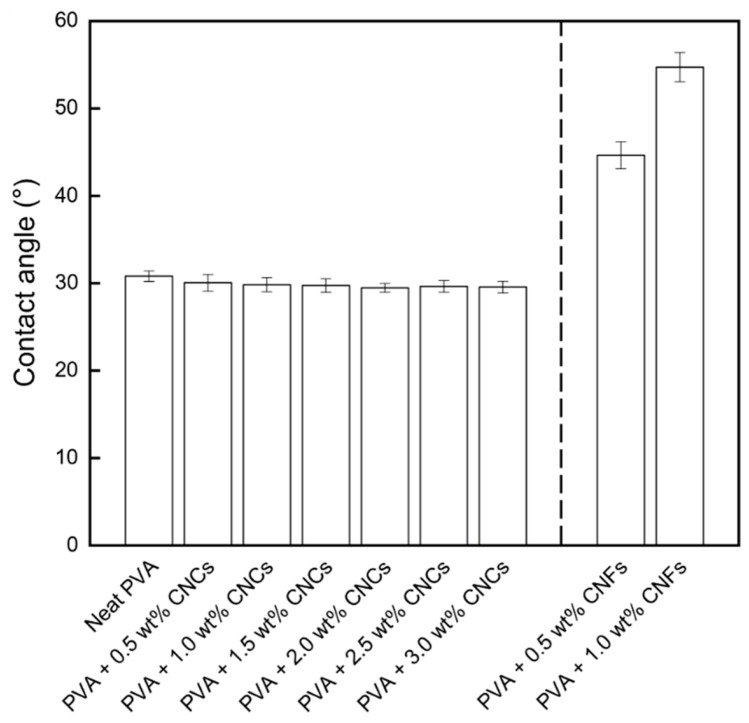
Contact angles of the PVA + CNC/CNF nanocomposite films.

The contact angles of the neat PVA and PVA + CNC/CNF nanocomposite films are shown in Figure 6. The contact angles of the CNC-incorporated PVA nanocomposite films were all slightly lower than that of neat PVA. This is because of the presence of sulfur trioxide (–SO_3_) functional groups on the CNC surfaces due to sulfuric acid hydrolysis, which can form ionic bonds with water. The electrostatic repulsion between these groups compensates for the adhesive force between PVA and the CNCs [33]. In the CNF-reinforced PVA nanocomposite films, the hydrogen bonds between the CNFs and PVA reduced the number of free hydroxyl groups of PVA, resulting in higher contact angles. Moreover, the CNF chains were entangled and formed a physical network, which moderately prevented water uptake [33].

#### 3.1.3. Water Vapor Permeability

**Figure 7:**
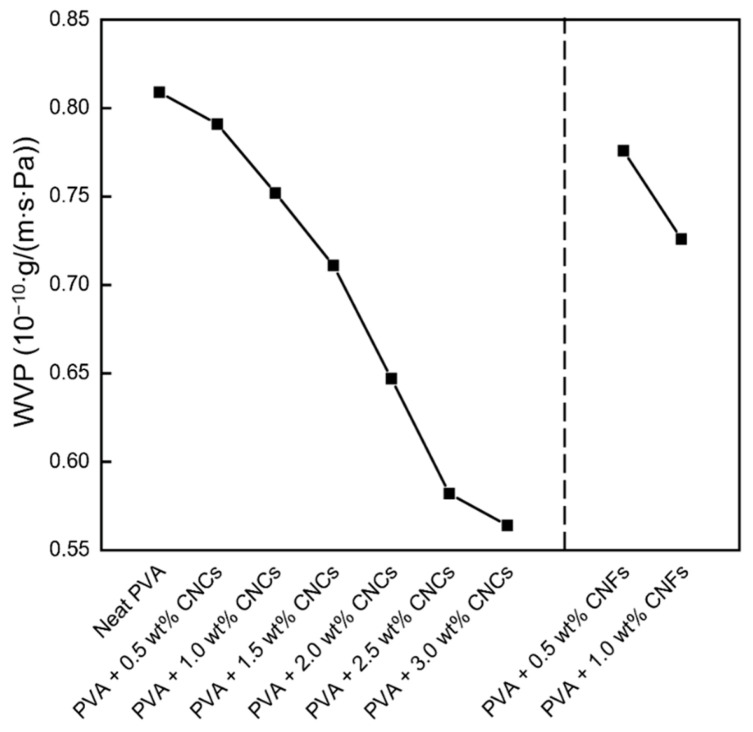
Water vapor permeabilities of the PVA + CNC/CNF nanocomposite films.

The WVP values of the neat PVA and PVA + CNC/CNF nanocomposite films are shown in Figure 7. The neat PVA film exhibited a high WVP (that is, poor barrier properties) owing to the numerous hydrogen bonds in its structure and its hydrophilic properties, which were confirmed by the contact angle tests. The addition of CNCs and CNFs to the films led to lengthening of the paths of water molecules permeating the PVA + CNC/CNF nanocomposite films [24], which reduced the WVP and improved the barrier properties of the films. The CNF-reinforced PVA composites exhibited lower WVPs compared to those of their CNC-reinforced equivalents, which was presumably because of the entanglement of CNF chains in the composite films, which forms a physical network that can somewhat prevent the penetration of water [33].

#### 3.1.4. Differential Scanning Calorimetry

**Figure 8:**
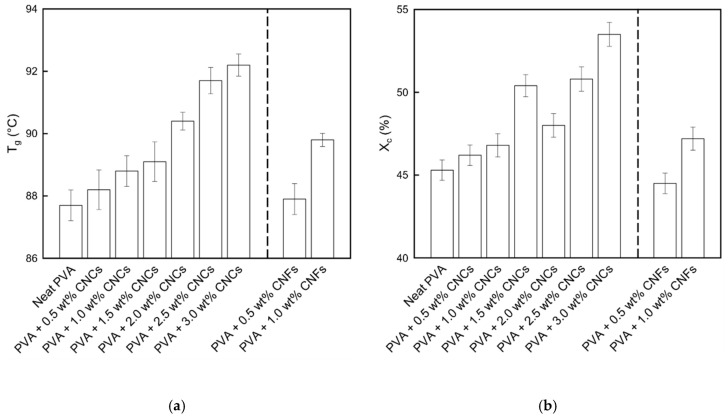
(**a**) T_g_ and (**b**) X_c_ values of the PVA + CNC/CNF nanocomposite films.

The T_g_ and X_c_ values of the neat PVA and PVA + CNC/CNF nanocomposite films are shown in Figure 8. The T_g_ was noted to slightly increase with the addition of CNCs or CNFs. This can be attributed to the strong interactions between the fillers and the substrate [44], including hydrogen bonds and van der Waals forces. Moreover, X_c_ exhibited a similar increasing trend, indicating that a large amount of energy was required to destroy the material. The nucleation caused by the addition of the nanocellulose led to the binding of numerous small crystal grains, which resulted in an increase in the crystal grain size (Figure 5).

#### 3.1.5. Thermogravimetric Analysis

**Figure 9:**
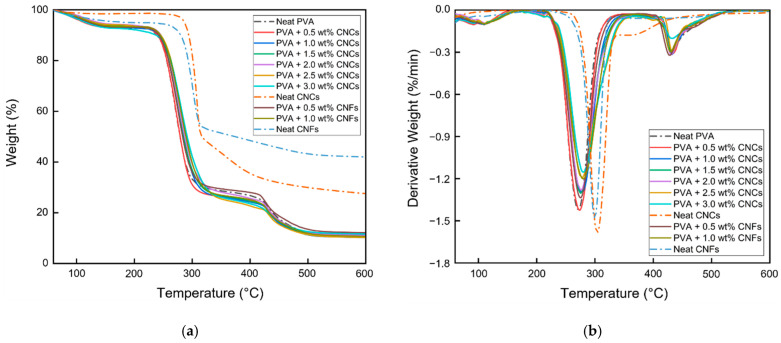
(**a**) TGA and (**b**) DTG profiles of the PVA + CNC/CNF nanocomposite films.

The TGA results of the neat PVA film, PVA + CNC/CNF nanocomposite films, neat CNCs, and neat CNFs are shown in Figure 9 and Table 3. Three mass loss regions were observed in the TGA data of the PVA + CNC/CNF nanocomposite films. The first region corresponds to water evaporation of the PVA + CNC/CNF nanocomposite films (60–150 °C) [45]. The second region corresponds to the degradation of the side chain (250 °C) and main chain (300 °C) of PVA [46], during which the nanocellulose also begins to degrade thermally (290–320 °C). The greatest mass loss was observed in this region (60–70%). At 350 °C, CNCs tend to get scorched owing to the presence of sulfate groups, whereas heating in air causes the oxidation of hydroxyl groups, resulting in the formation of free radicals [47]. CNFs, on the other hand, are composed of more amorphous regions, which enable their higher final contents. In the third region (>420 °C), the nanocellulose was decomposed into carbon dioxide, and the nanocellulose chain was depolymerized [48].

As shown in Figure 9b, the addition of CNCs or CNFs led to a deceleration of the degradation in the second region of the DTG curve; moreover, the DTG curve shifted toward the upper right. As shown in Table 3, T_onset_, T_offset_, and T_max_ exhibited increasing trends in the second region with the addition of CNCs or CNFs, suggesting that the addition of nanocellulose to PVA could improve its thermal properties.

### 3.2. Hydrophobic Materials

#### 3.2.1. Differential Scanning Calorimetry

**Figure 10:**
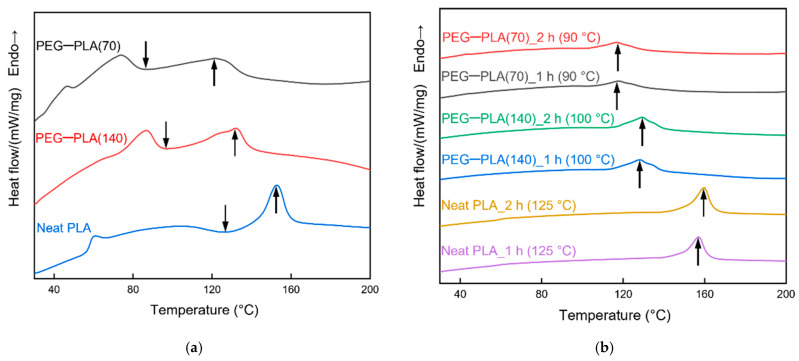
DSC analysis of the (**a**) untreated and (**b**) heat-treated hydrophobic materials.

DSC was performed to determine the heat-treatment temperature of the hydrophobic materials (Figure 10). The downward and upward arrows indicate T_cc_ and T_m_, respectively. Figure 10a shows the DSC results of the hydrophobic materials that were not subjected to heat treatment. The heating temperature must be higher than T_cc_ to induce significant crystallization in PLA; moreover, it should be lower than T_m_ to prevent melting of the material. However, the double-sided solution casting of neat PLA was performed during the preparation of the hydrophobic materials; therefore, neat PLA’s T_cc_ had to be considered. Therefore, the heat-treatment temperatures for PEG–PLA(70) and PEG–PLA(140) were set to 90 °C and 125 °C, and 100 °C and 125 °C, respectively; heat-treatment durations of 1 h and 2 h were used.

Figure 10b shows the DSC results of the heat-treated hydrophobic materials. The upward arrows indicate T_m_. The materials heat-treated for 1 h and 2 h exhibited similar T_m_ values; moreover, no obvious T_cc_ was observed. In particular, the crystallinity exhibited an increasing trend with increasing duration of the heat treatment, indicating that the heat treatment led to the crystallization of PLA. The specific values of crystallinity are presented in Table 4.

#### 3.2.2. Thermogravimetric Analysis

**Figure 11:**
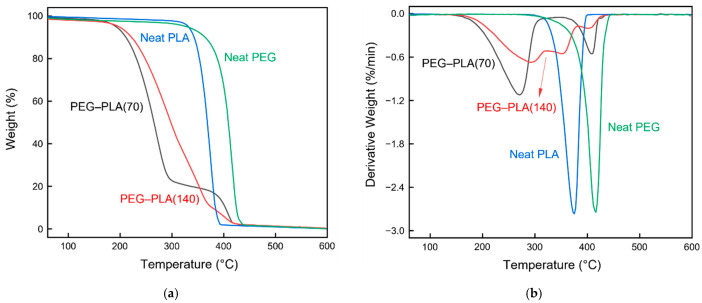
(**a**) TGA and (**b**) DTG profiles of the hydrophobic materials.

TGA was used to determine the possible thermal degradation of the hydrophobic materials caused by the heat treatment (Figure 11). Since the ether bonds in PEG–PLA are more stable than the ester groups, the ether bonds decompose at higher temperatures [49]. Two mass loss regions were observed for PEG–PLA, corresponding to the degradation of the ester groups of PLA (150–330 °C) and that of the ether bonds of PEG (345–430 °C), respectively. The thermal degradation of neat PLA can be attributed to the random main chain scission (350–400 °C) [50]. No substantial thermal degradation was observed upon treatment of the hydrophobic materials at 90, 100, and 125 °C (Figure 11). Therefore, these temperatures were feasible for heat treatment.

In the temperature range of 150–290 °C, PEG–PLA(70) exhibited a greater mass loss than that of PEG–PLA(140), which proved that the further addition of PLA could impede the thermal degradation of the material at these temperatures. However, the temperature increase in the 315–430 °C range led to PEG–PLA(70) exhibiting a smaller mass loss than that of PEG–PLA(140). Therefore, heat treatment was performed below the aforementioned temperature to prevent the significant thermal degradation of PLA at these temperatures.

#### 3.2.3. X-ray Diffraction

**Figure 12:**
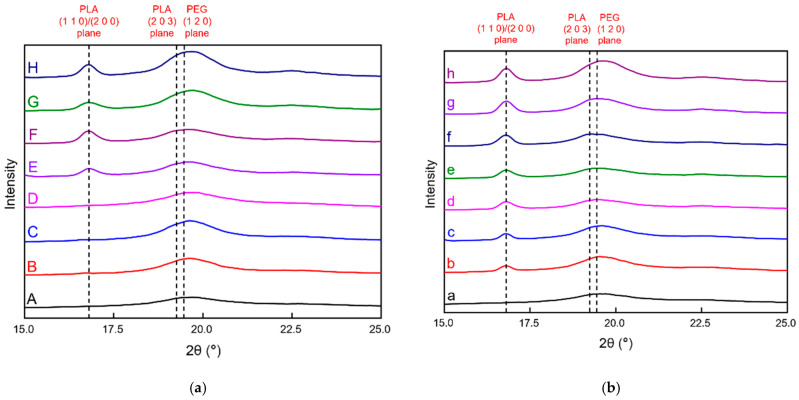
XRD patterns of untreated and heat-treated (**a**) PEG–PLA(70) and PEG–PLA(70) + neat PLA, and (**b**) PEG–PLA(140) and PEG–PLA(140) + neat PLA specimens. The temperatures, durations, and notations corresponding to this heat treatment are listed in Table 2.

The hydrophobic specimens prepared by double-sided solution casting were heat-treated at different temperatures and durations; the specific parameters are listed in Table 2. XRD analysis was performed to determine the presence of significant PLA crystallization (Figure 12). The PVA + 3 wt% CNCs film was used as the PVA + CNC/CNF nanocomposite film in this analysis.

Figure 12a shows the XRD patterns of sandwich-structured PVA-based PEG–PLA(70) and PEG–PLA(70) + neat PLA composite films subjected to different heat treatment conditions. After 1 h and 2 h of heat treatment of the PEG–PLA(70)-based films, only slight signals related to PLA and PEG were observed at 19.1° and 19.4°, which correspond to the (2 0 3) and (1 2 0) planes, respectively (B and C in Figure 12a) [51,52]. Although the DSC measurements indicated an upward trend of the crystallinity of PLA with heat treatment (Table 4), no significant PLA peak was detected. Comparison of the XRD patterns of untreated and heat-treated PEG–PLA(70) + neat PLA films (1 h and 2 h) reveals that the heat-treated materials exhibit obvious PLA and PEG peaks at 16.8°, 19.1°, and 19.4°, which correspond to the (1 1 0)/(2 0 0), (2 0 3), and (1 2 0) planes, respectively (E, F, G, and H in Figure 12a) [51,52,53], indicating that neat PLA undergoes significant crystallization at 90 °C and 125 °C. This observation agrees with the DSC results of the heat-treated materials, which exhibited relatively high PLA crystallinities (Table 4).

Figure 12b shows the XRD patterns of sandwich-structured PVA-based PEG–PLA(140) and PEG–PLA(140) + neat PLA composite films subjected to different heat treatment conditions. After 1 h and 2 h of heat treatment of the PEG–PLA(140)-based films, there are significant PLA and PEG signals at 16.8°, 19.1°, and 19.4°, which correspond to the (1 1 0)/(2 0 0), (2 0 3), and (1 2 0) planes, respectively (b and c in Figure 12b) [51,52,53]. This suggests the crystallization of the heat-treated PEG–PLA(140), which is consistent with the DSC measurements (Table 4). Comparison of the XRD patterns of the untreated and heat-treated PEG–PLA(140) + neat PLA specimens (1 h and 2 h) shows that the heat-treated materials exhibit obvious PLA and PEG peaks at 16.8°, 19.1°, and 19.4°, which correspond to the (1 1 0)/(2 0 0), (2 0 3), and (1 2 0) planes, respectively (e, f, g, and h in Figure 12b) [51,52,53], indicating the crystallization of the heat-treated PEG–PLA(140) and neat PLA. This result suggests that neat PLA exhibited significant crystallization at 100 °C and 125 °C, which agrees with the DSC results of the heat-treated materials that revealed the relatively high crystallinities of PLA (Table 4).

#### 3.2.4. Contact Angle Analysis

**Figure 13:**
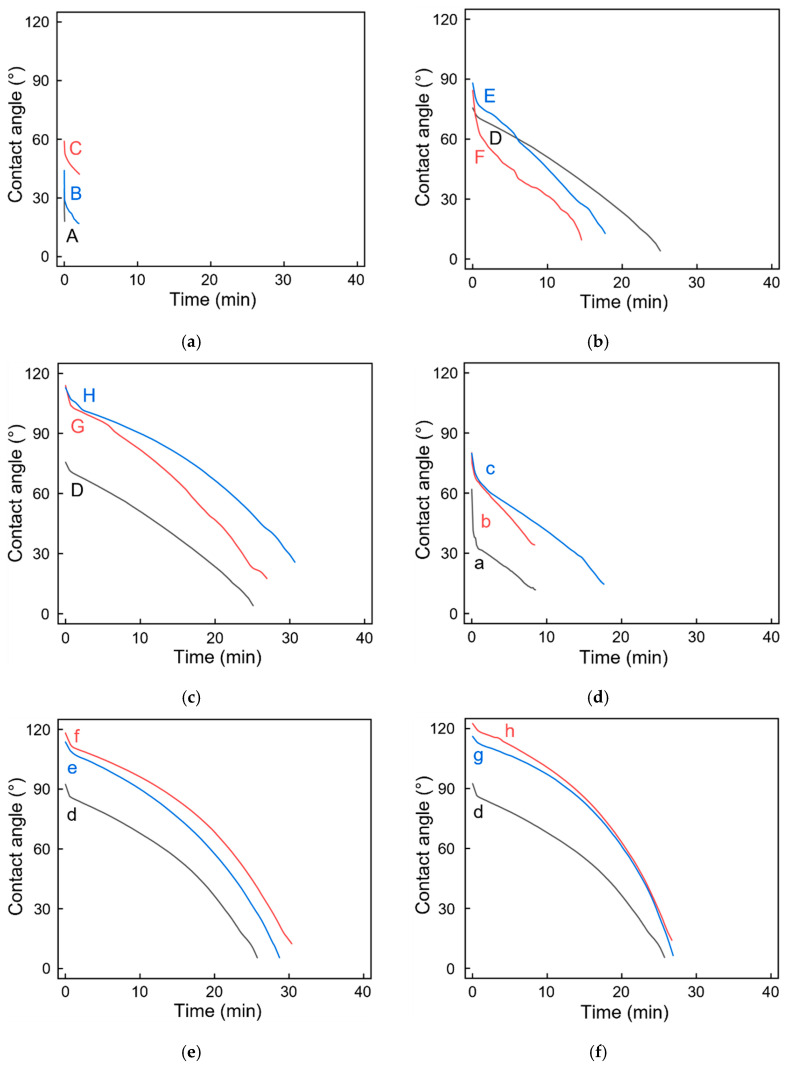
Contact angle analyses of untreated and heat-treated (**a**) PEG–PLA(70)_90 °C, (**b**) PEG–PLA(70) + neat PLA_90 °C, (**c**) PEG–PLA(70) + neat PLA_125 °C, (**d**) PEG–PLA(140)_100 °C, (**e**) PEG–PLA(140) + neat PLA_100 °C, and (**f**) PEG–PLA(140) + neat PLA_125 °C samples. The durations and notations corresponding to this heat treatment are shown in Table 2.

Long-term contact angles were measured to verify the possible effects of the presence of obvious PLA crystals on the hydrophilicity or hydrophobicity of the material surfaces (Figure 13). The PVA + 3 wt% CNCs film was used as the PVA + CNC/CNF nanocomposite film in this analysis.

Figure 13a shows the contact angles of the sandwich-structured PVA-based PEG–PLA(70) composite films with and without heat treatment at 90 °C. Increasing the duration of heat treatment led to an increase in the crystallization of PLA (Table 4); however, this trend was not obvious in the XRD results (B and C in Table 2). The contact angle and contact duration exhibited increasing trends at the beginning of the experiment; however, the contact angle rapidly decreased because of the presence of the hydrophilic PEG on the surface. Eventually, the sandwich-structured PVA-based PEG–PLA(70) composite films deformed because of water-droplet permeation. Figure 13b shows the contact angles of sandwich-structured PVA-based PEG–PLA(70) + neat PLA composite films with and without heat treatment at 90 °C. Since the T_cc_ of PEG–PLA(70) is 90 °C, which is 35 °C lower than the T_cc_ of PLA, PLA was not completely crystallized. However, PLA peaks clearly appeared in the XRD results (E and F in Table 2). Therefore, the heat-treated materials exhibited a relatively high initial contact angle; however, a rapid drop in the contact angle was observed because of the straightforward penetration of water droplets into PEG–PLA(70), which caused deformation of the film. Figure 13c shows the contact angles of sandwich-structured PVA-based PEG–PLA(70) + neat PLA composite films with and without heat treatment at 125 °C. In this case, the heat-treatment temperature increased to 125 °C, which reached the T_m_ of PEG–PLA(70) and the T_cc_ of PLA. Increasing the duration of the heat treatment led to an initial contact angle of nearly 120°; the contact duration was also the longest among all the PEG–PLA(70) + neat PLA specimens. This result demonstrated that even though the temperature of 125 °C had reached the T_m_ of PEG–PLA(70), its hydrophobic nature prolonged the duration of contact because of the obvious PLA peaks observed in the XRD results (G and H in Table 2).

Figure 13d shows the contact angles of sandwich-structured PVA-based PEG–PLA(140) composite films with and without heat treatment at 100 °C. Increasing the duration of heat treatment led to considerable increases in both the initial contact angle and contact duration compared with those of sandwich-structured PVA-based PEG–PLA(70) composite films with and without heat treatment at 90 °C (A, B, and C in Table 2). This is because PEG–PLA(140) contained twice as much PLA as PEG–PLA(70), indicating the presence of more PLA crystals in the latter; the contact angle decreased comparatively gradually. However, water droplets nonetheless permeated the sandwich-structured PVA-based PEG–PLA(140) composite films and eventually caused its deformation. Figure 13e shows the contact angles of the sandwich-structured PVA-based PEG–PLA(140) + neat PLA composite films with and without heat treatment at 100 °C. The T_cc_ of PEG–PLA(140) (100 °C) is 25 °C lower than that of neat PLA. However, the isothermal crystallization of neat PLA at 100 °C for 1 h can achieve a crystallinity close to 100% [54]. Therefore, the initial contact angles measured for the one-hour and two-hour-heat-treated specimens were similar and greater than 90°; moreover, the overall duration was the longest among all the PEG–PLA(140) + neat PLA specimens. Figure 13f shows the contact angles of the sandwich-structured PVA-based PEG–PLA(140) + neat PLA composite films with and without heat treatment at 125 °C. In this case, the temperature was increased to 125 °C, which was only 5 °C below the T_m_ of PEG–PLA(140). Although the materials were on the verge of melting, PLA crystals were still generated. For the specimens heat-treated for 1 h and 2 h, the initial contact angles were close to 120°, which reconfirmed the hydrophobicity of PLA at 125 °C. However, the overall duration was slightly shortened because PEG–PLA(140) approached its T_m_.

#### 3.2.5. Water Vapor Permeability

**Figure 14:**
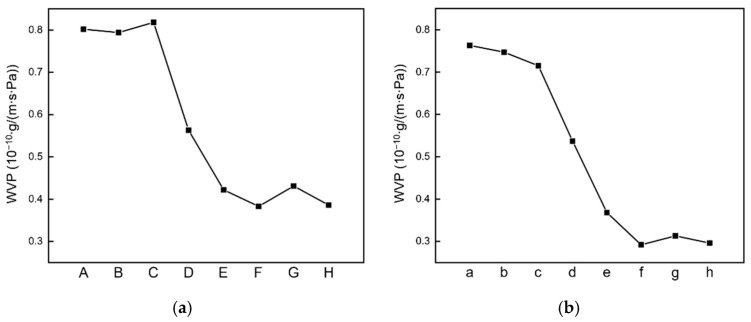
Water vapor permeabilities of the sandwich-structured PVA-based (**a**) PEG–PLA(70) and PEG–PLA(70) + neat PLA, and (**b**) PEG–PLA(140) and PEG–PLA(140) + neat PLA hydrophobic composite films. The temperatures, durations, and notations corresponding to this heat treatment are listed in Table 2.

In this experiment, the WVP was measured to determine the possible effects of the PLA crystals generated in the untreated and heat-treated specimens on the permeability of water vapor (Figure 14). The PVA + 3 wt% CNCs specimen was used as the PVA + CNC/CNF nanocomposite film in this analysis.

For the sandwich-structured PVA-based PEG–PLA(70) and PEG–PLA(140) composite film specimens without heat treatment prepared by double-sided solution casting, the addition of PEG–PLA lengthened the permeation path of water molecules through the PVA composite films. However, the presence of hydrophilic PEG on the surface eventually led to the permeation of the water vapor into the sandwich-structured PVA-based hydrophobic composite films and caused deformation. Hydrophobic PLA [55], on the other hand, exhibits excellent water vapor barrier properties, which can hinder the permeation of water vapor. With respect to the sandwich-structured PVA-based PEG–PLA(70) and PEG–PLA(140) composite film specimens with heat treatment, the presence of hydrophilic PEG on their surfaces led to water vapor permeation into the PVA composite film, although they exhibited relatively high X_c_(PLA) values (Table 4). However, the measured WVP exhibited a decreasing trend, indicating that the increase in X_c_ could enhance the water barrier performance of the material. The trend related to the decrease in WVP with an increase in X_c_ is similar to that observed in a CNC-incorporated PLA film by Luzi et al. [56].

With respect to sandwich-structured PVA-based PEG–PLA(70)/PEG–PLA(140) + neat PLA composite films, the heat treatment temperatures of 90 °C and 100 °C are the T_cc_ values of PEG–PLA(70) and PEG–PLA(140), respectively, both of which are lower than the T_cc_ of PLA. However, the isothermal crystallization of PLA indicates that it should be heated for 2 h at 90 °C or for ≈1 h at 100 °C to achieve a crystallinity close to 100% [54]. Therefore, excellent water barrier effects were observed. When the two materials were heat-treated at 125 °C, this temperature reached the T_m_ of PEG–PLA(70), which was close to the T_m_ of PEG–PLA(140) and identical to the T_cc_ of PLA. In this case, despite the heat-treated PLA having a higher X_c_, the water vapor barrier effects were slightly compromised because PEG–PLA(70) achieved a molten state and PEG–PLA(140) was close to it.

#### 3.2.6. X-ray Photoelectron Spectroscopy

**Figure 15:**
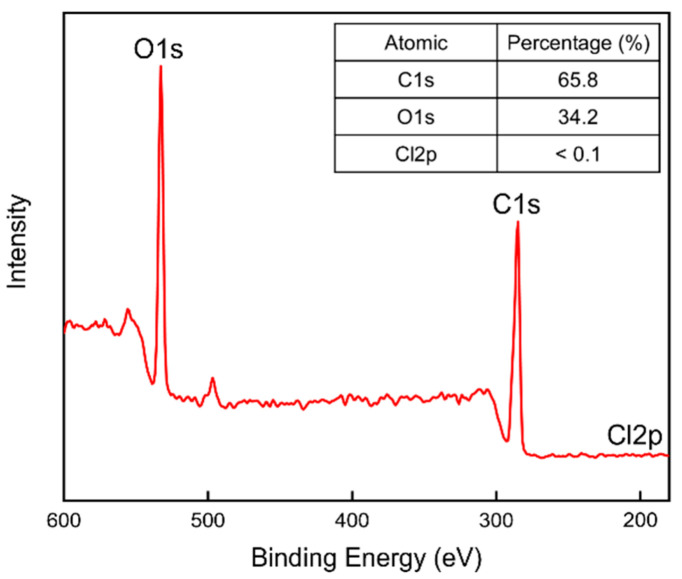
XPS profile of the sandwich-structured PVA-based hydrophobic composite film.

XPS analysis was performed to verify the presence of chlorine residues on the surfaces of the sandwich-structured PVA-based hydrophobic composite film. The PVA + 3 wt% CNCs film was used as the PVA + CNC/CNF nanocomposite film in this analysis. The PEG–PLA(140) + neat PLA film, which was prepared by double-sided solution casting and heat treatment for 2 h at 100 °C, was used as the hydrophobic film. Figure 15 shows the obtained XPS profile.

Carbon (C), oxygen (O), and chlorine (Cl) were selected for the composition analysis; the corresponding binding energies were 285.0, 531.0, and 198.5 eV, respectively. The table insert in Figure 15 indicates that almost no signal was detected for Cl, whose content was below 0.1%. This result confirms the evaporation of Cl during the preparation and the almost complete absence of Cl on the hydrophobic film, indicating the safety of the prepared film.

### 3.3. Heat-Treated Sandwich-Structured PVA-Based Hydrophobic Composite Film

#### 3.3.1. Contact Angle Analysis

**Figure 16:**
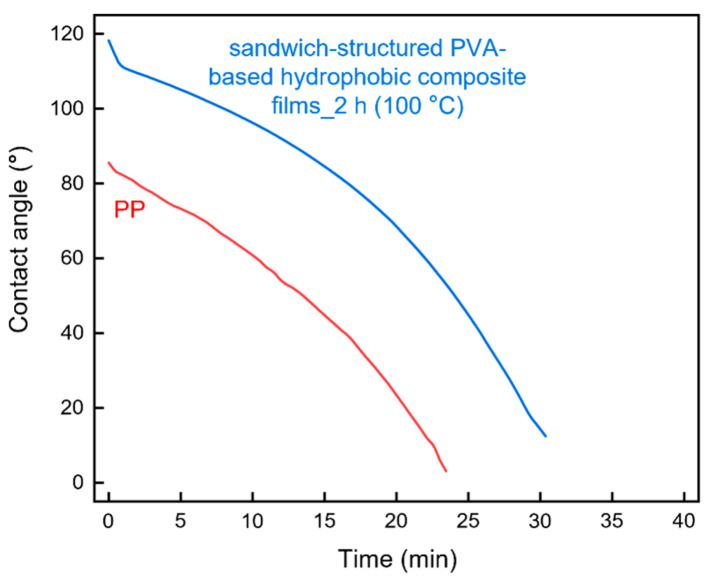
Contact angles of the sandwich-structured PVA-based hydrophobic composite film and commercially available PP.

The long-term contact angles of the heat-treated sandwich-structured PVA-based hydrophobic composite film and a commercial PP straw were compared (Figure 16). The PVA + 3.0 wt% CNCs specimen was used as the PVA + CNC/CNF nanocomposite film, and the PEG–PLA(140) + neat PLA specimen, which was prepared by double-sided solution casting and heat treatment for 2 h at 100 °C, was used as the hydrophobic film. Figure 16 shows that both the initial contact angle and the contact duration on the sandwich-structured PVA-based hydrophobic composite film were greater than those of the PP straw. However, the contact angles of both the materials decreased over time.

#### 3.3.2. Water Vapor Permeability

The WVP values of the heat-treated sandwich-structured PVA-based hydrophobic composite film and a commercial PP straw were compared (Table 5). The PVA + 3.0 wt% CNCs sample was used as the PVA + CNC/CNF nanocomposite film, and the PEG–PLA(140) + neat PLA sample, which was prepared by double-sided solution casting and heat treatment for 2 h at 100 °C, was used as the hydrophobic film. Since PP has a relatively large molecular weight (300,000–700,000) and a dense structure, it exhibited a relatively low WVP. In the case of the sandwich-structured PVA-based hydrophobic composite film, water gradually permeated into the hydrophobic film and accessed the PVA + CNC nanocomposite film, which absorbed the water; therefore, its WVP was higher than that of PP.

#### 3.3.3. Water Uptake

**Figure 17:**
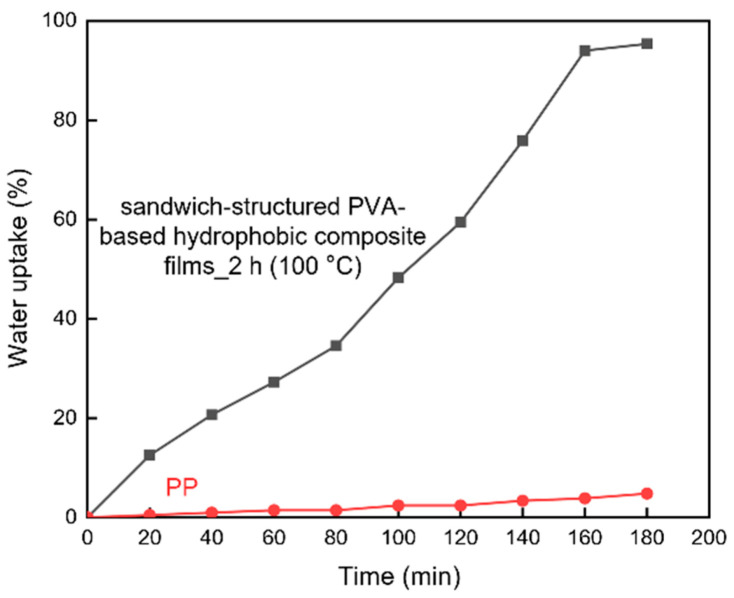
Water uptake behavior of the sandwich-structured PVA-based hydrophobic composite film and a commercial PP specimen.

Water uptake was measured to compare the swelling behavior of the heat-treated sandwich-structured PVA-based hydrophobic composite film and a commercial PP straw (Figure 17). The PVA + 3.0 wt% CNCs specimen was used as the PVA + CNC/CNF nanocomposite film, and the PEG–PLA(140) + neat PLA sample, which was prepared by double-sided solution casting and heat treatment for 2 h at 100 °C, was used as the hydrophobic film. Figure 17 indicates that the sandwich-structured PVA-based hydrophobic composite film exhibited a considerably higher water uptake compared to that of the PP sample. For similar reasons that were mentioned in Section 3.3.2 (WVP), water eventually permeates into the PVA + CNC nanocomposite film, which absorbs the water and swells. In the case of the PP sample, its high molecular weight hinders water permeation and swelling; therefore, only a slowly increasing trend was observed for its water uptake.

## 4. Conclusions

PVA + CNC/CNF nanocomposite films were prepared using blade coating and solution casting, and XRD analysis confirmed that these preparation processes did not damage the materials. The addition of CNCs or CNFs led to a decrease by up to 30% in the WVP because of an increase in the permeating path of water molecules through the PVA composite films; T_g_ and X_c_ also exhibited increasing 5% and 18%, respectively. Moreover, an increase in the crystal grain size was observed owing to the strong hydrogen bonds and van der Waals forces between the fillers and the substrate. TGA results showed that the T_onset_, T_offset_, and T_max_ parameters exhibited increases of up to 3%, 6%, and 3% in the second region, suggesting that the addition of the nanocellulose fillers could enhance the thermal properties of PVA.

Sandwich-structured PVA-based PEG–PLA + neat PLA composite films were prepared by double-sided solution casting to enhance the hydrophobicity of the PVA + CNC/CNF nanocomposite films. In addition, heat treatments at different temperatures and durations were conducted to increase the crystallinity of PLA, whose signals were observed in the XRD patterns. TGA analysis showed that the heat treatment did not cause substantial thermal degradation of the materials. The contact angles and WVPs of the heat-treated films were significantly improved compared to those of untreated PVA composite films owing to the hydrophobic nature of PLA. The residual amount of chlorine was also examined to establish the safety of the resulting films. The results showed that the chlorine content was less than 0.1%, suggesting its evaporation during the preparation and an insignificant presence on the surface of the hydrophobic films.

Finally, the sandwich-structured PVA-based hydrophobic composite film was compared with a commercial PP straw. Except for the long-term contact angle analysis, in which the sandwich-structured PVA-based hydrophobic composite film performed better than PP, the WVP and water uptake of the sandwich-structured PVA-based hydrophobic composite film were inferior to those of PP. This is because of the relatively large molecular weight and dense structure of PP. Therefore, the hydrophobic behavior of PVA is still lacking compared to that of commercial PP and requires further research. The sandwich-structured PVA-based hydrophobic composite film can be applied not only to the straws mentioned in this article but also to food packaging materials, such as sealing film and food preservation bags.

## Figures and Tables

**Table 1:** Properties of PEG–PLA used in the experiments.

**PEG–PLA(70)**			
**Material**	**Molecular Weight**	**Content**	**Equivalent**
PEG	2000	200 mg/0.1 mmol	1
LA	144.13	1008.91 mg/7 mmol	70
DBU	152.24	38.06 mg/0.25 mmol	2.5
**PEG–PLA(** **140)**			
**Material**	**Molecular Weight**	**Content**	**Equivalent**
PEG	2000	200 mg/0.1 mmol	1
LA	144.13	2017.82 mg/14 mmol	140
DBU	152.24	38.06 mg/0.25 mmol	2.5

**Table 2:** Temperatures, durations, and notations used for PEG–PLA heat treatment.

Hydrophobic Film Material		Heat Treatment		Notation
		Temperature (°C)	Time (h)	
PEG–PLA(70)	-	No Heat Treatment		A
		90	1	B
			2	C
	Neat PLA	No Heat Treatment		D
		90	1	E
			2	F
		125	1	G
			2	H
PEG–PLA(140)	-	No Heat Treatment		a
		100	1	b
			2	c
	Neat PLA	No Heat Treatment		d
		100	1	e
			2	f
		125	1	g
			2	h

**Table 3:** T_onset_, T_offset_, T_max_, and mass loss of the PVA + CNC/CNF nanocomposite films.

Sample	Second Region				Third Region			
	T_onset_ (°C)	T_offset_ (°C)	T_max_ (°C)	Mass Loss (%)	T_onset_ (°C)	T_offset_ (°C)	T_max_ (°C)	Mass Loss (%)
Neat PVA	248.8	292.5	271.2	64.1	419.4	460.4	432.1	17.9
PVA + 0.5 wt% CNCs	249.4	293.5	274.0	66.8	422.2	456.3	433.0	16.0
PVA + 1.0 wt% CNCs	250.6	301.1	277.0	67.5	423.3	450.1	432.0	15.5
PVA + 1.5 wt% CNCs	252.1	300.2	275.1	67.6	422.9	449.3	431.1	15.5
PVA + 2.0 wt% CNCs	253.1	300.9	276.1	66.7	422.1	448.9	430.1	16.4
PVA + 2.5 wt% CNCs	253.3	308.2	277.1	69.7	424.4	452.4	433.1	14.2
PVA + 3.0 wt% CNCs	256.4	309.2	280.0	66.1	418.9	462.4	433.0	13.5
Neat CNCs	297.3	312.8	304.2	52.8	322.0	404.4	357.2	11.2
PVA + 0.5 wt% CNFs	250.7	297.5	275.1	64.6	418.3	447.8	428.1	16.4
PVA + 1.0 wt% CNFs	252.9	306.8	280.1	67.0	421.4	448.1	430.1	15.8
Neat CNFs	284.6	310.8	299.2	43.6	324.5	503.6	419.2	9.4

**Table 4:** PLA crystallinities of heat-treated hydrophobic materials.

Hydrophobic Materials	Heat Treatment	X_c_ (PLA) (%)
PEG–PLA(70)	-	5.1
	1 h (90 °C)	24.1
	2 h (90 °C)	28.9
PEG–PLA(140)	-	1.1
	1 h (100 °C)	37.9
	2 h (100 °C)	39.0
Neat PLA	-	16.5
	1 h (125 °C)	52.2
	2 h (125 °C)	62.4

**Table 5:** WVP of the sandwich-structured PVA-based hydrophobic composite film and a commercially available PP straw.

Material	WVP (10^−10^ g/(m·s·Pa))
PP	0.005
sandwich-structured PVA-based hydrophobic composite film	0.292

## Data Availability

The data presented in this study are available on request from the corresponding author.

## Appendix A

**Table A1:** List the abbreviations used in this study.

Abbreviation	Definition	Abbreviation	Definition
CNCs	Cellulose nanocrystals	PEG–PLA	Polyethylene-glycol–poly(lactic acid)
CNFs	Cellulose nanofibers	PP	Polypropylene
DCM	Dichloromethane	PVA	Polyvinyl alcohol
DSC	Differential scanning calorimetry	ROP	Ring-opening polymerization
DTG	Derivative thermogravimetry	TGA	Thermogravimetric Analysis
EA	Ethyl acetate	WVP	Water vapor permeability
GO	Graphene oxide	WVTR	Water vapor transmission rate
LA	Lactide	XPS	X-ray photoelectron spectroscopy
LDPE	Low-density polyethylene	XRD	X-ray diffraction
